# Associations between lifestyle and initiation of cardiovascular preventive medicines in 60-year-olds at low to moderate cardiovascular risk level – a prospective cohort study based on the VIPVIZA-trial

**DOI:** 10.1186/s12872-026-05922-6

**Published:** 2026-05-07

**Authors:** Tove Lindström Lundgren, Eva-Lotta Glader, Henrik Holmberg, Margareta Norberg, Anders Själander, Eva Sönnerstam

**Affiliations:** 1 Department of Medical and Translational biology, Umeå University, Umeå, 901 87 Sweden; 2 Department of Public Health and Clinical Medicine, Umeå University, Umeå, 901 87 Sweden; 3 Department of Epidemiology and Global Health, Umeå University, Umeå, 901 87 Sweden

**Keywords:** lifestyle, adherence, antihypertensives, lipid-lowering medicines, cardiovascular prevention, diet, exercise

## Abstract

**Background:**

Medicines complement healthy lifestyles in cardiovascular disease (CVD) prevention, but knowledge is lacking on how behaviours in these domains are related. This study aimed at investigating associations between physical activity and diet, and time to initiation of preventive medicines.

**Method:**

A cohort study based on the “Visualization of Asymptomatic Atherosclerotic Disease for Optimal Cardiovascular Prevention” (VIPVIZA)-trial, including 60-year-olds of low/moderate CVD-risk who were treatment-naive to antihypertensives or lipid-lowering medicines six months prior trial-inclusion. Sex-specific Cox proportional hazards models were used to analyse impacts of diet and physical activity, each categorized in three levels (least healthy – moderately healthy – healthiest), on initiation of antihypertensives and lipid-lowering medicines.

**Results:**

Of 1891 individuals in the cohort, 1340 (58% women) were antihypertensive-naive and 1783 (57% women) were lipid-lowering naive. Significantly faster initiation of antihypertensives was seen in women with the healthiest diet (HR [95%CI] 2.46 [1.18–5.14], ref. least healthy) whereas higher levels of physical activity were significantly associated with slower initiation of antihypertensives in women (HR [95%CI], moderate; 0.51 [0.30–0.88]), high; 0.48 [0.30–0.77], ref. low). Neither diet nor physical activity levels affected initiation of lipid-lowering medicines in women, and lifestyle factors were not associated with initiation of antihypertensives or lipid-lowering medicines in men.

**Conclusions:**

Relationships between lifestyle and medicine initiation varies with health behavior, drug-class, and sex. Regarding diet, initiation of antihypertensives in women may agree with a healthy adherer effect, whereas for physical activity, the association appear inverse. Diet and physical activity levels are less influential for initiation in men, and for lipid-lowering medicines. As findings are uncertain, further studies are needed to clarify relationships between these factors.

**Trial registration:**

VIPVIZA-trial registration number: NCT01849575, registration date: 2013-05-08.

**Supplementary Information:**

The online version contains supplementary material available at 10.1186/s12872-026-05922-6.

## Background

Cardiovascular disease (CVD)-risk is affected by modifiable lifestyle factors such as diet and physical activity. Additionally, antihypertensives and lipid-lowering medicines are effective and safe preventive treatments, and guidelines advocate for a complementary approach between lifestyle modification and medicines in CVD-prevention [1].

Whether pharmacological or lifestyle related, the success of any preventive treatment is dependent on adherence. Initiation being the first step, followed by implementation. Given that lifestyle can modify CVD-risk, a healthy lifestyle has been associated with less use [2] and delayed initiation [3], of CVD-preventive medicines. However, health-seeking patients may also be more inclined or urged to initiate preventive drug therapy, and patients who initiate and adhere to one preventive therapy may be more likely to engage in other healthy behaviours as well, referred to as healthy user or adherer effects [4–6]. In contrast, attitudes of medicines replacing lifestyle, or vice versa, may also prevail. Possibly explained by risk offsetting/compensative reasoning [7], some may consider medicines as easier options, preferable to making lifestyle changes [8]. Perhaps more common, patients and prescribers may feel that lifestyle changes alone should be tried for a period, before initiation of drug treatment is considered [9–11].

Risk assessment models accounting for the multifactorial nature of CVD, such as Systemic Coronary Risk Estimation (SCORE) [12] and updated SCORE2 [1, 13], are used to guide physicians in best preventive treatment. However, guidelines emphasize individualized assessments when considering drug treatment for risk factors, especially so in individuals at low to moderate cardiovascular risk, and treatment decisions should always be based on shared decision-making [1]. As inadequate risk factor control as well as under-prescribing of CVD-preventive medicines have been reported [14–16], there is evidently a need for improved preventive strategies. One effort, targeted at patients and physicians, and which the present study is based on, was the population-based Pragmatic Randomized Open Blinded End-point trial (PROBE), Visualization of Asymptomatic Atherosclerotic Disease for Optimal Cardiovascular Prevention, nested in an established community intervention programme, the Västerbotten Intervention Programme (VIPVIZA, VIP) [17]. VIPVIZA utilized a multifactorial intervention based on results of a carotid ultrasound examination, results of the trial have been reported previously [18–25]. In short, decelerated CVD-risk factor burden [18, 19], increased prescribing and faster initiation of statins [21, 22], as well as better adherence to prescribed statins [23], were seen in the intervention-group. The intervention also affected participants’ risk perceptions [20], and evoked cognitive and emotional responses which were positively associated with lifestyle modification [25].

Improvements are needed in CVD-prevention, both regarding the proper use of medicines and in motivating healthier lifestyles [14]. As such, knowledge of how healthy or unhealthy behaviours relate to the initiation of CVD-preventive medicines may help guide future interventions targeted at lifestyle as well as pharmacological treatment. The objective of the present study is therefore to investigate if lifestyle factors, namely physical activity and diet, are associated with time to initiation of CVD-preventive medicines for primary prevention in a low to moderate risk population. In accordance with healthy user/adherer effects, our hypothesis is that healthy lifestyle factors are positively associated with initiation of CVD-preventive medicines.

## Methods

### Study setting: VIP and VIPVIZA overview

In Västerbotten County, Sweden, risk factor screening, management and health counselling is offered to residents the year they turn 40, 50 and 60 via a prevention programme, the VIP [17]. VIPVIZA was a pragmatic trial, additional to the VIP. Within the VIP-cohort, between 2013 and 2016, participation in VIPVIZA was offered to 40-year-olds with a first degree relative with a history of CVD before 60 years age, 50-year-olds with one or more designated risk factor; smoking, diabetes, hypertension, low density lipoprotein-cholesterol (LDL-C) >174 mg/dL, abdominal obesity, and 60-year-olds irrespective of additional inclusion criteria. Eligible, consenting individuals were randomized to the VIPVIZA intervention or control group after completing baseline evaluations in the VIP. Thereafter, a first VIPVIZA-visit was scheduled.

The VIPVIZA-intervention was based on carotid ultrasound examinations. In the intervention-group, ultrasound results were presented to participants and their general practitioners as a simplified picture of carotid arteries, a graphic depiction on the presence or absence of plaque and an estimation of the individuals vascular age in relation to their chronological age. Participants were also informed of atherosclerosis being a dynamic process, modifiable by lifestyle and preventive medicines, and received a follow-up phone call from a nurse educated in motivational interviewing. Both intervention and control group participants received care according to clinical guidelines for cardiovascular risk management at their respective health care centres as offered within the already established prevention programme [17]. Detailed descriptions on recruitment and the intervention are available in previous publications [18–20].

### Study variables and outcomes

#### Physical activity level

Participants’ commuting activity, physical activity at work, leisure activities and physical exercise, were evaluated by questionnaire at the baseline VIP-visit [17]. The time spent performing activities of different intensities was used to assign participants as belonging to one of three categories of physical activity: (1) “Low” (sedentary/very low activity), (2) “Moderate” (moderately intensive activity < 150 min/week) and (3) “High/recommended” (moderately intensive activity > 150 min/week or highly intensive activity > 75 min/week). These categories were defined based on guideline recommendations for physical activity.

#### Diet

Dietary habits were collected at baseline using a Food Frequency Questionnaire (FFQ), previously validated by recall interviews and dietary biomarkers in the VIP-cohort [26–28]. The FFQ inquires frequencies of consumption of 64 food items on a nine-level scale, ranging from “never” to “4 or more times a day” [29]. Responders estimate their portion sizes for staple food, meat and vegetables, portion sizes for other foods are determined using sex- and age-specific average portion sizes from a validation study in a subset of VIP participants, or using data from the Swedish Food Agency [27].

A Healthy Diet Score (HDS), as described previously [30], but specifically adapted to the FFQ in VIP, was used in the present study. In short, to generate the HDS, food items are categorized as favourable (fish, fruits, vegetables, whole grains) or unfavourable (red or processed meats, desserts/sweets, sugar-sweetened beverages, fried potatoes). Intake frequencies of the food groups are ranked in each sex, for favourable food groups in ascending quartiles from 0 to 3 (0 = lowest intake, 3 = highest intake), for unfavourable food groups in descending quartiles (3 = lowest intake, 0 = highest intake). Summation of quartiles generates a score ranging from 0 to 24, where higher scores indicate a healthier diet with higher intake of favourable food groups and lower intake of unfavourable food groups [29].

Participants were classified in three dietary categories: 1) “Least healthy” (HDS 0–8), 2) “Moderately healthy” (HDS 9–16), 3) “Healthiest” (HDS 17–24). Based on the assumption that higher HDS-scores reflect higher adherence to a healthy diet, but in absence of a standardized interpretation of HDS comparable to that available for physical activity level, the total range (0–24) was divided into approximate thirds to define these categories. Whilst a score of 0 is theoretically possible it was not observed in the VIPVIZA population, and because HDS scores are integers, i.e. three exactly equal score groups cannot be created, the “Least Healthy” (0–8) category was decided to span across 9 points, whereas the other two each cover 8 points. For the entire cohort, scores were symmetrically distributed, the cut-offs represent 70% of participants belonging in the “Moderately Healthy” category, and 15% in the lower (0–8) and higher (17-24) ranges.

#### Cardiovascular risk level

SCORE [12], which accounts for sex, age, smoking status, total cholesterol and systolic blood pressure (SBP), was used to characterize participants’ cardiovascular risk level at baseline, as SCORE2 was not developed at the time of recruitment. Low risk was defined as < 1% 10-year risk of fatal CVD, and moderate as 1–4% 10-year risk of fatal CVD.

#### Outcomes

The study outcomes were initiation of antihypertensives and lipid-lowering medicines. Initiation was defined as a first dispensation occurring after inclusion in the VIPVIZA-trial, until the participant’s three-year follow-up visit. Individual dispensing-data was collected from the National Prescribed Drug Register at the National Board of Health and Welfare, Sweden. Records were retrieved starting from six months prior to the initial ultrasound visit to decide treatment-naivety. Antihypertensives were defined as belonging to Anatomic Therapeutic Chemical classification system (ATC)-codes C03A, C07, C08 and C09, lipid-lowering medicines as belonging to ATC-code C10.

### Population

The cohort consisted of VIPVIZA-participants fulfilling the inclusion criteria: (1) being of low or moderate SCORE-risk, (2) being treatment-naive to antihypertensives and/or lipid-lowering medicines, (3) being 60 years old, (4) having completed questionnaires regarding ≥ 1 of physical activity level and dietary habits. Exclusions based on SCORE-risk levels (high or very high) were justified by the study aims. Only 60-year-old participants were included because they were not subjected to any inclusion criteria aside from age, in contrast, 40- and 50-year-olds were offered participation in VIPVIZA based on risk factors or family history, thus limiting their representativeness.

### Statistical analyses

IBM^®^ SPSS^®^ Statistics Version 29.0.1.0 was used for statistical analyses. Baseline characteristics: SCORE, physical activity level, diet, body mass index (BMI), blood pressure and blood lipids, were summarized by frequencies, percentages and mean values with standard deviations. Differences in the proportions of categorical variables; diet, physical activity level, BMI and smoking, between sexes were assessed using chi-square tests, regardless of whether the variables were ordinal. Trend analyses were not performed. For means (blood pressure, blood lipids), differences were evaluated by independent sample t-tests.

Effects of diet and physical activity level, on time to initiation of antihypertensives and lipid-lowering medicines were evaluated by cox regression. As sex and gender differences in risk factors, development, treatment and prevention of CVD are known [31], analyses were stratified by women/men. Due to the study setting within the VIPVIZA-trial, and a presumed influence of risk level on initiation, models were adjusted for these factors by including randomization-group and SCORE-risk level as covariables. Models were not explicitly adjusted for the constitutes of SCORE, i.e. blood lipid levels, blood pressure or smoking, to avoid overfitting whilst keeping models similar in the adjusted analyses of antihypertensives as well as lipid-lowering medicines initiation. To assess whether BMI confounds associations between diet, physical activity and initiation, models adjusted for BMI (categorized as < 25, 25–30, > 30) were fitted. Further, sensitivity analyses where SCORE risk levels were replaced with systolic blood pressure for antihypertensive initiation and LDL-C for lipid lowering initiation were also conducted, as were analyses using continuous HDS. Missing values were not imputed; all analyses were based on complete cases only. Alpha was 0.05 for all statistical analyses, with no adjustment for multiple comparisons. Results are presented as hazard ratios (HR) with 95% confidence intervals (CI).

### Ethical Considerations

The VIPVIZA-trial was conducted according to ethical principles based on the Helsinki Declaration, Good Clinical Practice and national regulatory instructions. The trial was approved by the Regional Ethical Review Board at Umeå University (Dnr 2011-445-31 M). Participants received written and oral information including declarations of participation being voluntary and the ability to withdraw at any time without consequences. Written informed consent was obtained from all participants.

## Results

### Population

**Fig. 1:**
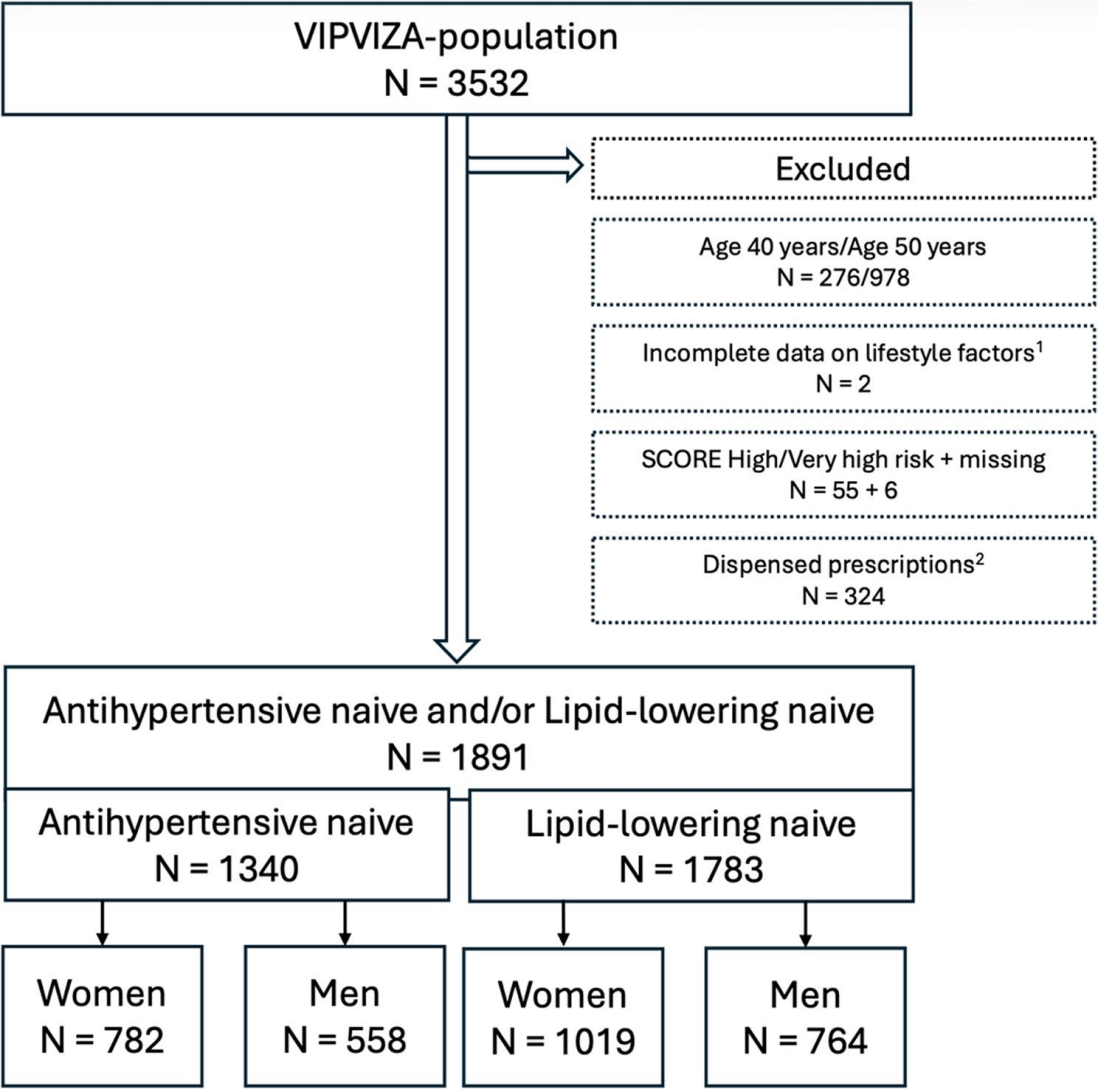
Flow chart illustrating how the population eligible for analysis was arrived at. The total antihypertensive/lipid-lowering naïve population represents individuals naïve to treatment with either or both drug-classes, as such the same participant can be included in both the antihypertensive and the lipid-lowering naive subpopulations. ^1^Did not answer diet or physical activity questionnaires. ^2^Dispensed prescriptions of antihypertensive and lipid-lowering medicines within 6 months prior to baseline.

In total, there were 1891 participants of 60 years age, naive to treatment with antihypertensives and/or lipid-lowering medicines, of low to moderate SCORE-risk level. A flow chart of the arrival at the analysed cohort is shown in Fig. 1.

Seven hundred-and-eighty-two women (49.1% in intervention group) and 558 men (49.8% in intervention group) were naive to treatment with antihypertensives (Table 1). Corresponding numbers of lipid-lowering naive individuals were 1019 women (48.8% in intervention group) and 764 men (48.8% in intervention group). In women 75.2% of antihypertensive naive, and 71.1% of lipid-lowering naive participants were of low SCORE-risk. In men, 98.6% of antihypertensive naïve, and 99.3% of lipid-lowering naïve participants were of moderate SCORE-risk. Most participants reported reaching high/recommended levels of physical activity (56.8% − 61.6%), no significant differences in physical activity level between sexes were observed. In both sexes and in both groups of medicine-naivety, the majority reported a moderately healthy diet (68.7% − 73.2%). Between sexes, the proportions in other dietary categories (least healthy and healthiest) were similar in antihypertensive naive participants, whereas a larger proportion of lipid-lowering naive women (17.3%) reported the healthiest diet compared to men (11.9%). There were overall differences between men and women in all isolated risk factors, reflective of the higher risk level in men, except for LDL-C (Table 1).

**Table 1:** Baseline characteristics of study cohort

N (%)	Antihypertensive naive		Lipid-lowering naive	
	Women	Men	Women	Men
	782	558	1019	764
Physical Activity	P* = 0.677		P* = 0.216	
Low level	107 (13.9)	81 (14.7)	151 (15.0)	116 (15.3)
Moderate level	188 (24.4)	144 (26.1)	245 (24.4)	211 (27.9)
High level	474 (61.6)	327 (59.2)	608 (60.6)	430 (56.8)
missing	13	6	15	7
Diet	P* = 0.148		P* = 0.011	
Least healthy	104 (14.0)	71 (13.3)	136 (14.0)	113 (15.7)
Moderately healthy	512 (68.7)	390 (73.2)	666 (68.7)	520 (72.2)
Healthiest	129 (17.3)	72 (13.5)	168 (17.3)	87 (12.1)
missing	37	25	49	44
BMI	P* < 0.001		P* < 0.001	
< 25 kg/m^2^	373 (47.7)	197 (35.3)	430 (42.2)	230 (30.1)
25–30 kg/m^2^	254 (32.5)	274 (49.1)	368 (36.1)	382 (50.1)
> 30 kg/m^2^	155 (19.8)	87 (15.6)	221 (21.7)	151 (19.8)
missing	0	0	0	1
HDL-C	P^ < 0.001		P ^ < 0.001	
mean (SD), mmol/L	1.64 (0.45)	1.29 (0.35)	1.61 (0.44)	1.27 (0.34)
LDL-C	P^ = 0.344		P ^= 0.385	
mean (SD), mmol/L	3.75 (0.91)	3.70 (0.93)	3.71 (0.85)	3.67 (0.86)
Total-C	P^ < 0.001		P^ < 0.001	
mean (SD), mmol/L	5.95 (0.98)	5.64 (1.01)	5.90 (0.91)	5.60 (0.92)
Systolic BP	P^ < 0.001		P^ < 0.001	
mean (SD), mmHg	124.83(15.07)	128.54 (13.32)	127.84 (15.97)	131.00 (14.03)
Diastolic BP	P^ < 0.001		P^ < 0.001	
mean (SD), mmHg	79.00 (9.05)	81.69 (8.67)	80.58 (9.45)	83.70 (9.49)
Smoking	P*= 0.188		P* = 0.001	
Yes	92 (11.8)	53 (9.5)	130 (12.8)	60 (7.9)
No	690 (88.2)	405 (90.5)	889 (87.2)	704 (92.1)
SCORE	P* < 0.001		P * < 0.001	
Low	588 (75.2)	8 (1.4)	725 (71.1)	5 (0.7)
Moderate	194 (24.8)	550 (98.6)	294 (28.9)	759 (99.3)

*From Pearson chi-square; ^two-sided p t-test for equality of means; *SCORE* Systematic coronary risk estimation, *BMI* Body mass index, *HDL* high density lipoprotein, *LDL* low density lipoprotein, *C* Cholesterol, *BP* blood pressure

### Initiation of preventive medicines

**Fig. 2:**
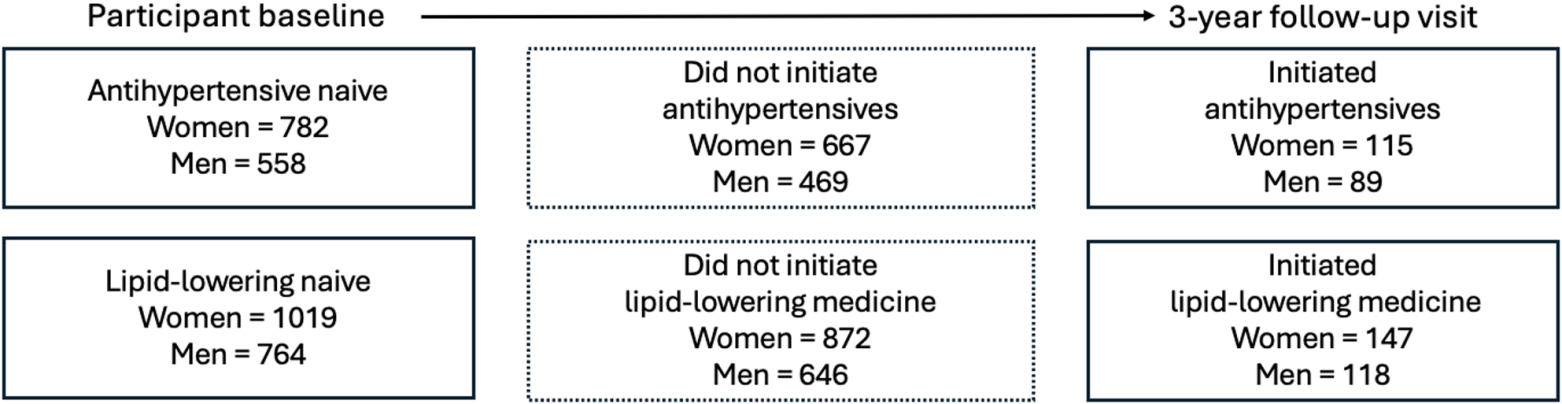
Shows the number of cohort participants in each group of medicine naivety who initiated medicines during the study period

Of the 782 antihypertensive naïve women, 115 initiated treatment within the three years of follow-up, whereas the number of women who initiated lipid-lowering treatment was 147 of 1019 (Fig. 2). As presented in Tables 2 and 3, in women, having the healthiest eating habits was significantly associated with faster initiation of antihypertensive medicines (Adjusted HR [95% CI], 2.46 [1.18–5.14], ref. least healthy). When HDS was treated as continuous, the effect of healthier diet on initiation of antihypertensives in women was however not significant (HR [95% CI], 1.05 [0.999–1.1], Additional file 1, Table A6). High or moderate physical activity levels in women were significantly associated with slower (Adjusted HR [95% CI]; high; 0.48 [0.30–0.77] moderate; 0.51 [0.30–0.88], ref. low) initiation of antihypertensive medicines (Tables 2 and 4). No significant associations between lifestyle factors and initiation of lipid-lowering medicines were seen in women (Tables 2, 3 and 4).

In men, 89 of 558 antihypertensive naïve, and 118 of lipid-lowering naïve participants initiated treatment during the follow-up period (Fig. 2). There were no significant associations between initiation of antihypertensives or lipid-lowering medicines and lifestyle habits in crude (Table 2) or adjusted models (Tables 3 and 4).

The intervention was associated with significantly faster initiation of lipid-lowering medicines, but not antihypertensives, in women and men (Tables 3 and 4). Moderate SCORE-risk, compared to low, was associated with faster initiation of antihypertensives, but not lipid-lowering medicines in women (Tables 3 and 4). There were no initiators among men of low SCORE risk level, resulting in disproportionately large HRs and wide CIs for moderate SCORE risk level in men. To assess if the low number of low-risk men were influential for the results of diet and physical activity, additional models excluding low-risk men were tested. Results remained similar in these models (not shown). Replacing SCORE risk level with SBP or LDL-C did not alter results regarding diet or physical activity level and initiation in either sex, neither did adding BMI to models (results available in Additional File 1). Slower initiation of antihypertensives in women with moderate physical activity was however not significant in models adjusted for SBP and BMI (Additional file 1, Table A3). When treated as a continuous measure, increasing HDS was not associated with initiation in any of the models (Additional file 1, Table A6).

**Table 2:** Initiation of medicines in 60-year-old women and men of low to moderate SCORE risk. HR (95% CI), from unadjusted cox regression analyses

Crude HR (95% CI)	Women		Men	
	Antihypertensive	Lipid-lowering	Antihypertensive	Lipid-lowering
Diet ref Least Healthy				
Moderately healthy	1.60 (0.83–3.10)	1.55 (0.89–2.71)	1.12 (0.57–2.18)	1.30 (0.74–2.29)
Healthiest	**2.08 (1.00-4.34)**	1.29 (0.66–2.51)	1.43 (0.64–3.22)	1.48 (0.71–3.06)
N events/tot	112/745^1^	139/970^3^	85/533^5^	111/720^7^
Physical activity ref Low				
Moderate	**0.53 (0.31–0.91)**	1.12 (0.67–1.87)	1.51 (0.70–3.26)	1.19 (0.66–2.15)
High/recommended	**0.48 (0.30–0.76)**	0.90 (0.56–1.42)	1.61 (0.80–3.25)	1.12 (0.65–1.94)
N events/tot	111/769^2^	146/1004^4^	88/552^6^	117/757^8^
Randomization ref Control				
Intervention	0.84 (0.58–1.22)	**3.73 (2.55–5.45)**	1.11 (0.73–1.68)	**2.53 (1.72–3.74)**
N events/tot	115/782	147/1019	89/558	118/764
SCORE				
ref Low			N events in ref. category =0	N events in ref. category = 0
Moderate	**2.46 (1.70–3.56)**	1.21 (0.86–1.70)	20.56 (0.01-3.8 × 10^4^ )	20.30 (0.00-3.0 × 10^5^)
N events/tot	115/782	147/1019	89/558	118/764

*HR* hazard ratio, *SCORE* Systematic Coronary Risk Estimation, ^1^Missing values for diet = 37, ^2^Missing values for Physical activity = 13, ^3^Missing values for diet = 49, ^4^Missing values for Physical activity = 15, ^5^Missing values for diet = 25, ^6^Missing values for Physical activity = 6, ^7^Missing values for diet = 44, ^8^Missing values for Physical activity = 7. Significant effects are presented in bold

**Table 3:** Initiation of medicines in 60-year-old women and men of low to moderate SCORE risk. HR (95% CI), from cox regression analyses of diet, adjusted for SCORE risk level and randomization-group

Adjusted HR (95% CI)	Women		Men	
	Antihypertensive	Lipid-lowering	Antihypertensive	Lipid-lowering
Diet ref Least Healthy				
Moderately healthy	1.75 (0.90–3.39)	1.56 (0.89–2.73)	1.11 (0.57–2.17)	1.24 (0.70–2.19)
Healthiest	**2.46 (1.18–5.14)**	1.39 (0.71–2.72)	1.45 (0.64–3.26)	1.44 (0.69–2.98)
Randomization ref Control				
Intervention	0.83 (0.57–1.20)	**3.73 (2.52–5.51)**	1.05 (0.69–1.61)	**2.52 (1.69–3.76)**
SCORE				
Ref low			N events in ref category = 0	N events in ref category = 0
Moderate	**2.72 (1.87–3.96)**	1.24 (0.87–1.77)	6.35 × 10^4^(0.00-1.08 × 10^183^)	2.18 × 10^4^ (0.00-1.6 × 10^146^)
N events/tot	112/745^1^	139/970^2^	85/533^3^	111/720^4^

*HR* hazard ratio, *SCORE* Systematic Coronary Risk Estimation, ^1^Missing values for diet = 37, ^2^Missing values for diet = 49, ^3^Missing values for diet = 25, ^4^Missing values for diet = 44. Significant effects are presented in bold

**Table 4:** Initiation of medicines in 60-year-old women and men of low to moderate SCORE risk. HR (95% CI), from cox regression analyses of physical activity, adjusted for SCORE risk level and randomization-group

Adjusted HR (95% CI)	Women		Men	
	Antihypertensive	Lipid-lowering	Antihypertensive	Lipid-lowering
Physical activity ref Low				
Moderate	**0.51 (0.30–0.88)**	1.10 (0.66–1.82)	1.54 (0.71–3.33)	1.24 (0.68–2.25)
High/recommended	**0.48 (0.30–0.77)**	0.91 (0.57–1.45)	1.63 (0.81–3.30)	1.12 (0.65–1.94)
Randomization ref Control				
Intervention	0.82 (0.57–1.20)	**3.66 (2.51–5.36)**	1.06 (0.70–1.61)	**2.49 (1.68–3.67)**
SCORE				
ref Low			N events in ref category = 0	N events in ref category = 0
Moderate	**2.46 (1.69–3.58)**	1.17 (0.83–1.66)	6.56 × 10^4^ (0.00-1.79 × 10^184^)	2.17 × 10^4^ (0.00-1.53 × 10^142^)
N events/tot	111/769^1^	146/1004^2^	88/552^3^	117/757^4^

*HR* hazard ratio, *SCORE* Systematic Coronary Risk Estimation, ^1^Missing values for Physical activity = 13, ^2^Missing values for Physical activity = 15, ^3^Missing values for Physical activity = 6, ^4^Missing values for Physical activity = 7. Significant effects are presented in bold

## Discussion

Initiation of medicines in relation to lifestyle factors, in a low and moderate CVD-risk population, has to our knowledge not been studied previously. As such, this study contributes new knowledge about behaviours in these domains. Most of the cohort were of high physical activity level (~ 60%), only a minority reported having the healthiest diet (~ 15%) (Table 1). Whereas women with the *healthiest* diet were *faster* initiators of antihypertensives than women with the least healthy diet, women reporting *higher* levels of physical activity instead initiated these medicines slower, compared to women with low levels of physical activity. In men there were no significant effects of diet or physical activity level on initiation of either drug-class.

Previously, Ribo-Coll et al. found a healthy lifestyle (considering diet and exercise) to delay *initiation* of cardiovascular preventive medicines in an older population of higher CVD-risk [3]. Prevalent antihypertensive and lipid-lowering medicine users have however been found to engage in healthy behaviours to greater extent compared to non-users [32]. Other studies, by Aggarwal and Mosca [33], as well as Lee et al. [6], have found positive associations between medication *adherence* and other healthy behaviours, whereas Nakajima et al. [34] reported the opposite. In the present study, some indications of overall faster initiation with healthier habits, in accordance with healthy adherer effects, were seen. However, an inverse association was evident for women regarding physical activity level and antihypertensives. Thus, if accounting for previous studies as well as the present, relationships between medicine use and lifestyle factors appear complex and somewhat inconclusive.

An interesting aspect of the present study is the influence of the physicians, as they are responsible for issuing the prescription which sets the starting point from when initiation is possible. During the study period, European guidelines recommended lifestyle changes for weeks/months prior to initiation of antihypertensives in low/moderate risk individuals [35, 36]. Viewing results from the opposite perspective means women with the least healthy diets were significantly slower initiators of antihypertensives. This could reflect a willingness towards endorsing/opting for dietary changes over immediate initiation of medicines, in line with guidelines as well as with some patients’ and physicians’ preferences expressed in previous research [8, 10]. Applying similar reasoning for physical activity in women however, the faster initiation in women with low physical activity level would then instead indicate a preference for immediate initiation of medicines over trying lifestyle modification first. This would be more in line with attitudes of medicines replacing lifestyle, or a preference for medication over lifestyle changes that have been described before [7, 8]. These results are also aligned with other studies reporting overall higher use of prescribed medications with lower levels of physical activity [37, 38]. It is noteworthy that high physical activity level has previously been shown to attenuate the risk of hypertension in obese women [39], whereas in women of the present study, lower physical activity level was associated with higher BMI, albeit not to blood pressure (Additional file 1, table A7). Whilst the associations between physical activity and initiation of antihypertensives were not changed by adjusting for BMI, it is possible that initiation to some extent was propelled by a combination higher BMI and sedentary lifestyle.

Previously, women and men have been found to differ in dietary habits and physical activity level [40, 41], as well as adherence to, and attitudes towards medicines [42]. The proportions of women and men with healthier or less healthy behaviours were similar in the present study. Lifestyle however seemed of greater importance for the initiation of antihypertensives in women considering no significant effects were seen in men. That most analysed men were of moderate SCORE-risk level, whereas most women were of low, could explain this. Possibly, lifestyle factors are most influential for initiation in lower risk individuals, where risk reduction is likely perceived as less urgent.

There are some explanations to why no significant associations between lifestyle factors and initiation of lipid-lowering medicines were seen in either sex. For starters, attitudes towards, and adherence to, a medicine, can differ between drug classes [43]. Further, guideline recommendations for lipid-lowering medicines state that if plaques are present, initiation is indicated, whereas for antihypertensives, presence of plaque do not solely constitute an indication [1]. This renders the VIPVIZA-intervention by design as more likely to affect initiation of lipid-lowering medicines. Indeed, the VIPVIZA-intervention have previously been shown to increase prescribing, initiation of, and adherence to, lipid-lowering medicines, without effect on antihypertensives [22, 23], and the intervention was strongly associated with faster initiation of only lipid-lowering medicines also in the present study (Tables 2 and 3). As such, the risk of residual confounding by the intervention effect resulting from treating the VIPVIZA-population as a cohort is likely greater when lipid-lowering medicines are concerned, i.e., it is probable that the intervention effect was superior to influences of current lifestyle on the initiation of lipid-lowering medicines.

This study is not without limitations. Firstly, no multiplicity adjustments were made, and as ∼1–2 findings can be expected to be positives by chance, results should be interpreted as exploratory. It should also be noted that selecting different cut-offs for the diet score used in this study could have produced other results. The effect of diet on initiation of antihypertensives in women can thus be considered as particularly uncertain, especially given that in sensitivity analyses using a continuous score, diet was not significant. Moreover, the low number of events limited the possibility to account for confounders, and interactions between study variables were not tested. Whereas associations were not changed by adding BMI or substituting SCORE for SBP or LDL-C, interactive effects and other factors such as socioeconomic status (SES) may have influenced results. For instance, disparities in lifestyle and health depending on SES are well documented [44], and lower SES can be a risk factor for hypertension [45] as well as medication non-adherence [46]. To capture the complex interrelationships and interactions between all these factors was beyond the scope of the present study, yet the authors recognize this as a limitation to consider when interpreting the results. Other limitations are the small number of men with low SCORE risk, that prescriber and patient effects on outcomes are intertwined, and that indications for starting or not starting treatment are unknown. Moreover, the use of self-reported lifestyle factors may have introduced recall or social desirability bias. Lastly, the analyses did not consider changes from baseline regarding the studied lifestyle factors, and the categorization of health behaviours intro discrete levels inevitably entails a loss of nuance. All limitations considered, further research is needed to corroborate and elaborate findings of the present study. Future studies should focus on improved control for factors mentioned above or be designed to examine their interrelationships and interactions. Study strengths include the comprehensive dispensing data from the National Prescribed Drug Register, which includes all pharmacy dispensed prescriptions in Sweden. That the 60-year-old participants were not subjected to any eligibility criteria other than age is another strength, contributing to sample representativeness. However, generalizability is still limited to settings with similar preventive strategies, and to individuals of comparable age.

## Conclusions

The relationship between lifestyle and medicine initiation varies with health behaviour, drug-class, and sex. Regarding dietary habits, initiation of antihypertensives in women may agree with a healthy adherer effect, whereas in the case of physical activity, the association appear inverse. In men, and for lipid-lowering medicines, dietary habits and physical activity levels seem less influential for initiation. However, these findings are uncertain, warranting further research to clarify relationships among lifestyle factors and initiation of preventive medicines.

## Supplementary Information

Supplementary Material 1

## Data Availability

The data underlying this article is owned by Umeå University and was provided by the VIPVIZA project group by permission. Data will be shared on reasonable request to the PI of VIPVIZA: Patrik Wennberg (Email: patrik.wennberg@umu.se).

## Abbreviations

ATC: Anatomic Therapeutic Chemical classification system
BMI: Body Mass Index
BP: Blood Pressure
CI: Confidence Interval
CVD: Cardiovascular Disease
FFQ: Food Frequency Questionnaire
HDS: Healthy Diet Score
HR: Hazard Ratio
LDL: Low Density Lipoprotein
PROBE: Pragmatic Randomized Blinded End–point trial
SBP: Systolic Blood Pressure
SCORE: Systematic Coronary Risk Estimation
SES: Socioeconomic status
SPSS: Statistical Package for the Social Sciences
VIP: Västerbotten Intervention Programme
VIPVIZA: Västerbotten Intervention Programme, Visualization of Asymptomatic Atherosclerotic Disease for Optimal Cardiovascular Prevention
