# Supplementary material for: Associations between lifestyle and initiation of cardiovascular preventive medicines in 60-year-olds at low to moderate cardiovascular risk level – a prospective cohort study based on the VIPVIZA-trial
Source: BMC Cardiovasc Disord. 2026 May 7;26:412. doi: 10.1186/s12872-026-05922-6 (PMC13162362; doi:10.1186/s12872-026-05922-6)
Supplement: Supplementary file 1 — Supplementary Material 1 [file 12872_2026_5922_MOESM1_ESM.docx]

Additional File 1

Table of Contents

[Table A1. Initiation of medicines in 60-year-old men and women of low to moderate SCORE risk. HR (% CI) from unadjusted cox regression analyses of BMI, SBP and LDL-C. 2](#_Toc223514359)

[Table A2. Initiation of medicines in 60-year-old men and women of low to moderate SCORE risk. HR (95% CI) from cox regression analyses of Diet, adjusted for SBP (antihypertensives) or LDL-C (lipid-lowering medicines), BMI and randomization-group. 2](#_Toc223514360)

[Table A3. Initiation of medicines in 60-year-old men and women of low to moderate SCORE risk. HR (95% CI) from cox regression analyses of Physical activity, adjusted for SBP (antihypertensives) or LDL-C (lipid-lowering medicines), BMI and randomization-group. 3](#_Toc223514361)

[Table A4. Initiation of medicines in 60-year-old women and men of low to moderate SCORE risk. HR (95% CI) from cox regression analyses of Diet, adjusted for SCORE risk level, BMI and randomization-group. 4](#_Toc223514362)

[Table A5. Initiation of medicines in 60-year-old women and men of low to moderate SCORE risk. HR (95% CI) from cox regression analyses of Physical activity, adjusted for SCORE risk level, BMI and randomization-group. 5](#_Toc223514363)

[Table A6. Initiation of medicines in 60-year-old women and men of low to moderate SCORE risk. HR (95% CI) from cox regression analyses using continuous HDS, adjusted for SCORE risk level and randomization group. 5](#_Toc223514364)

[Table A7. Median (IQR) for SBP, LDL-C and BMI (continuous) in antihypertensive and lipid-lowering naïve women and men of different Physical Activity and Diet categories. 6](#_Toc223514365)

# Table A1. Initiation of medicines in 60-year-old men and women of low to moderate SCORE risk. HR (% CI) from unadjusted cox regression analyses of BMI, SBP and LDL-C.

| Crude HR (95% CI) | Women | | Men | |
| --- | --- | --- | --- | --- |
|  | Antihypertensive | Lipid-lowering | Antihypertensive | Lipid-lowering |
| BMI ref <25 25-30 >30  N events/tot | **1.58 (1.03-2.44)**  **2.10 (1.32-3.32)**  115/782 | 1.34 (0.93-1.93)  1.06 (0.68-1.65)  147/872 | **1.89 (1.13-3.18)**  **2.34 (1.25-4.39)**  89/558 | **2.04 (1.26-3.30)**  **1.92 (1.09-3.38)**  118/763 |
| SBP | **1.06 (1.04-1.07)**  115/782 | 1.00 (0.99-1.01)  147/1019 | **1.06 (1.05-1.08)**  89/558 | 1.01 (0.995-1.02)  118/764 |
| LDL-C | 0.94 (0.77-1.15)  113/776 | **1.84 (1.53-2.20)**  144/1013 | 0.96 (0.76-1.20)  89/555 | **1.57 (1.27-1.94)**  116/759 |

*BMI, Body mass index; SBP = Systolic blood pressure; LDL-C, low density lipoprotein cholesterol*

# Table A2. Initiation of medicines in 60-year-old men and women of low to moderate SCORE risk. HR (95% CI) from cox regression analyses of Diet, adjusted for SBP (antihypertensives) or LDL-C (lipid-lowering medicines), BMI and randomization-group.

| Adjusted HR (95% CI) | Women | | Men | |
| --- | --- | --- | --- | --- |
|  | Antihypertensive | Lipid-lowering | Antihypertensive | Lipid-lowering |
| Diet  ref Least Healthy Moderately healthy Healthiest | 1.74 (0.90-3.36)  **2.64 (1.26-5.52)** | 1.70 (0.95-3.03) 1.58 (0.79-3.16) | 0.88 (0.45-1.74) 1.10 (0.48-2.52) | 1.02 (0.58-1.80)  1.32 (0.61-2.74) |
| Randomization ref Control Intervention | 0.87 (0.60-1.27) | **3.18 (2.13-4.75)** | 1.14 (0.74-1.75) | **2.44 (1.62-3.67)** |
| BMI ref <25 25-30 >30 | 1.27 (0.82-1.97)  1.40 (0.87-2.26) | 1.47 (1.01-2.16)  1.31 (0.81-2.11) | 1.41 (0.83-2.39) 1.37 (0.70-2.65) | **2.15 (1.30-3.54)**  **2.88 (1.59-5.19)** |
| SBP | **1.06 (1.04-1.07)** | X | **1.06 (1.04-1.08)** | X |
| LDL-C | X | **1.49 (1.24-1.79)** | X | **1.51 (1.20-1.91)** |
| N events/tot | 112/745 | 136/956 | 85/533 | 109/714 |

*BMI, Body mass index; SBP, Systolic blood pressure; LDL-C, low density lipoprotein cholesterol; X, Not tested*

# Table A3. Initiation of medicines in 60-year-old men and women of low to moderate SCORE risk. HR (95% CI) from cox regression analyses of Physical activity, adjusted for SBP (antihypertensives) or LDL-C (lipid-lowering medicines), BMI and randomization-group.

| Adjusted HR (95% CI) | Women | | Men | |
| --- | --- | --- | --- | --- |
|  | Antihypertensive | Lipid-lowering | Antihypertensive | Lipid-lowering |
| Physical activity ref Low Moderate High/recommended | 0.59 (0.34-1.02)  **0.57 (0.36-0.91)** | 1.10 (0.65-1.88)  0.86 (0.53-1.41) | 1.44 (0.66-3.11) 1.53 (0.75-3.11) | 1.37 (0.75-2.48)  1.18 (0.68-2.06) |
| Randomization ref Control Intervention | 0.85 (0.59-1.24) | 3.18 (2.15-4.69) | 1.13 (0.74-1.72) | **2.37 (1.59-3.53)** |
| BMI ref <25 25-30 >30 | 1.15 (0.74-1.80)  1.17 (0.72-1.90) | 1.42 (0.98-2.07)  1.24 (0.78-1.99) | 1.40 (0.82-2.37) 1.40 (0.73-2.66) | **2.14 (1.31-3.49)**  **2.61 (1.47-4.67)** |
| SBP | **1.05 (1.04-1.07)** | X | **1.06 (1.04-1.08)** | X |
| LDL-C | X | **1.49 (1.24-1.78)** | X | **1.43 (1.14-1.80)** |
| N events/tot | 111/769 | 143/989 | 88/552 | 115/751 |

*BMI, Body mass index; SBP, Systolic blood pressure; LDL-C, low density lipoprotein cholesterol; X, not tested*

# Table A4. Initiation of medicines in 60-year-old women and men of low to moderate SCORE risk. HR (95% CI) from cox regression analyses of Diet, adjusted for SCORE risk level, BMI and randomization-group.

| Adjusted HR (95% CI) | Women | | Men | |
| --- | --- | --- | --- | --- |
|  | Antihypertensive | Lipid-lowering | Antihypertensive | Lipid-lowering |
| Diet  ref Least Healthy Moderately healthy Healthiest | 1.76 (0.91–3.41)  **2.59 (1.24–5.42)** | 1.58 (0.90-2.76)  1.39 (0.71-2.73) | 1.09 (0.56-2.12)  1.57 (0.69-3.54) | 1.21 (0.68-2.13)  1.48 (0.72-3.08) |
| Randomization ref Control Intervention | 0.84 (0.58–1.22) | **3.74 (2.53-5.53)** | 1.06 (0.69-1.62) | **2.52 (1.69-3.76)** |
| SCORE  Ref low Moderate | **2.50 (1.71–3.67)** | 1.23 (0.86-1.76) | N events in ref category =0  6.24x10^4^(0.00-3.42x10^183^) | N events in ref category =0  5.04x10^4^ (0.00-2.25x10^216^) |
| BMI ref <25 25-30 >30 | 1.46 (0.94-2.26)  **1.85 (1.15-2.97)** | 1.39 (0.96-2.02)  1.00 (0.62-1.61) | **1.89 (1.12-3.19)**  **2.28 (1.19-4.36)** | **2.09 (1.27-3.41)**  **2.07 (1.16-3.71)** |
| N events/tot | 112/745 | 139/970 | 85/533 | 111/719 |

*BMI, Body mass index; SCORE, Systematic Coronary Risk Estimation*

# Table A5. Initiation of medicines in 60-year-old women and men of low to moderate SCORE risk. HR (95% CI) from cox regression analyses of Physical activity, adjusted for SCORE risk level, BMI and randomization-group.

| Adjusted HR (95% CI) | Women | | Men | |
| --- | --- | --- | --- | --- |
|  | Antihypertensive | Lipid-lowering | Antihypertensive | Lipid-lowering |
| Physical activity ref Low Moderate High/recommended | **0.53 (0.31-0.91)**  **0.52 (0.33–0.84)** | 1.10 (0.66-1.84)  0.69 (0.57-1.46) | 1.54 (0.71-3.33)  1.63 (0.81-3.30) | 1.28 (0.71-2.33)  1.12 (0.69-2.07) |
| Randomization ref Control Intervention | 0.83 (0.57-1.20) | **3.69 (2.52-5.39)** | 1.07 (0.71-1.63) | **2.54 (1.72-3.75)** |
| SCORE  ref Low Moderate | **2.31 (1.58–3.39)** | 1.17 (0.82-1.66) | N events in ref category =0  6.41x10^4^ (0.00-9.37x10^184^) | N events in ref category = 0  5.04x10^4^ (0.00-5.15x10^214^) |
| BMI ref <25 25-30 >30 | 1.33 (0.85-2.07)  1.58 (0.97-2.56) | 1.36 (0.94-1.95)  1.02 (0.64-1.62) | **1.94 (1.15-3.27)**  **2.45 (1.30-4.61)** | **2.11 (1.31-3.42)**  **1.92 (1.08-3.39)** |
| N events/tot | 111/769 | 146/1004 | 88/552 | 117/756 |

*BMI, Body mass index; SCORE, Systematic Coronary Risk Estimation*

# Table A6. Initiation of medicines in 60-year-old women and men of low to moderate SCORE risk. HR (95% CI) from cox regression analyses using continuous HDS, adjusted for SCORE risk level and randomization group.

| Adjusted | Women | | Men | |
| --- | --- | --- | --- | --- |
|  | Antihypertensive | Lipid-lowering | Antihypertensive | Lipid-lowering |
| Diet  HDS (Range 0-24) | 1.05 (0.999–1.1) | 1.01 (0.97-1.05) | 1.00 (0.95-1.06) | 1.03 (0.98-1.08) |
| Randomization ref Control Intervention | 0.82 (0.57–1.19) | **3.74 (2.53-5.52)** | 1.05 (0.69–1.61) | **2.50 (1.68–3.73)** |
| SCORE  Ref low Moderate | **2.69 (1.84–3.92)** | 1.23 (0.87–1.76) | 6.14x10^4^(0.00-2.13x10^183^) | 2.12x10^4^ (0.00-1.15x10^146^) |
| N events/tot | 112/745 | 139/970 | 85/533 | 111/720 |

*HDS, Healthy Diet Score; SCORE, Systematic Coronary Risk Estimation*

# Table A7. Median (IQR) for SBP, LDL-C and BMI (continuous) in antihypertensive and lipid-lowering naïve women and men of different Physical Activity and Diet categories.

|  | **Median (IQR)** | | | **P*** |
| --- | --- | --- | --- | --- |
| **Physical Activity level** | **Low** | **Moderate** | **High Level** |  |
| **SBP (mmHg)** | | | | |
| AH-Naive Women | 124.00 (23.00) | 125.00 (22.00) | 124.00 (20.75) | 0.552 |
| AH-Naive Men | 127.00 (16.00) | 130.00 (18.75) | 128.00 (18.00) | 0.792 |
| **LDL-C (mmol/L)** | | | | |
| LL-Naive Women | 3.60 (1.10) | 3.70 (1.20) | 3.70 (1.10) | 0.095 |
| LL-Naive Men | 3.70 (1.20) | 3.70 (1.11) | 3.60 (1.10) | 0.555 |
| **BMI (kg/m^2^)** | | | | |
| AH-Naive Women | 27.34 (6.90) | 26.97 (6.80) | 24.49 (5.06) | <0.001 |
| AH-Naive Men | 27.31 (4.57) | 26.23 (4.38) | 25.76 (3.71) | 0.021 |
| LL-Naive Women | 28.40 (7.22) | 27.38 (6.39) | 25.22 (5.37) | <0.001 |
| LL-Naive Men | 27.47 (4.91) | 26.84 (4.83) | 26.22 (4.11) | 0.002 |
| **Diet** | **Least healthy** | **Moderately healthy** | **Healthiest** |  |
| **SBP (mmHg)** | | | | |
| AH-Naive Women | 125.00 (21.00) | 124.00 (22.00) | 123.00 (16.00) | 0.403 |
| AH-Naive Men | 127.00 (20.00) | 129.00 (18.00) | 127.50 (18.75) | 0.259 |
| **LDL-C C (mmol/L)** | | | | |
| LL-Naive Women | 3.60 (1.00) | 3.70 (1.10) | 3.80 (1.12) | 0.678 |
| LL-Naive Men | 3.55 (1.17) | 3.70 (1.10) | 3.60 (1.30) | 0.119 |
| **BMI (kg/m^2^)** | | | | |
| AH-Naive Women | 25.92 (5.42) | 25.23 (6.57) | 24.86 (5.15) | 0.107 |
| AH-Naive Men | 25.83 (4.71) | 26.18 (4.31) | 25.28 (3.80) | 0.088 |
| LL-Naive Women | 26.96 (5.99) | 25.84 (6.45) | 25.22 (5.35) | 0.074 |
| LL-Naive Men | 26.45 (4.76) | 26.59 (4.43) | 25.42 (3.51) | 0.029 |

*IQR, interquartile range; SBP, Systolic blood pressure; LDL-C, Low density lipoprotein cholesterol; BMI, Body mass index; AH, Antihypertensive; LL, Lipid-lowering, *Independent samples Kruskal-Wallis Test*
